# Effect of Diluents and Storage Time on the Cryopreservation of Collared Peccary (*Pecari tajacu*) Semen after Cooling Storage in a Transport Container at 5 °C

**DOI:** 10.3390/ani14060934

**Published:** 2024-03-18

**Authors:** Romário P. Santos, Andréia M. Silva, Ana G. Pereira, Yasmim C. S. Cavalcante, Yuri G. Matos, Gabriel S. C. Bezerra, Lilian L. Dantas, Alexandre R. Silva

**Affiliations:** Laboratory of Animal Germplasm Conservation, Department of Animal Sciences, Federal University of Semiarid Region–UFERSA, Mossoró 59625-900, RN, Brazil; romario.parente@hotmail.com (R.P.S.); andreia.m.silva@hotmail.com (A.M.S.); anagloriavet@gmail.com (A.G.P.); yacs.cavalcante@gmail.com (Y.C.S.C.); yurigmatos@gmail.com (Y.G.M.); gabrielscbezerra@gmail.com (G.S.C.B.); liliandantas29@gmail.com (L.L.D.)

**Keywords:** tayassuids, biobanking, Botutainer^®^, PRIMXcell Ultra^®^, semen transport

## Abstract

**Simple Summary:**

Given the persistence of certain threats to the survival of several free-living species, the need for efficient strategies for the conservation of genetic resources becomes increasingly urgent. An excellent option to help improve cryopreservation in peccaries would be to combine it with specific protocols and equipment for semen cooling, allowing transport and cooling curves different from those already used in conventional cryopreservation protocols. The ability to transport refrigerated semen from free-living species would allow the exchange of valuable biological material between institutions (zoos, reserves, breeding facilities, etc.), followed by shipment to a specialized laboratory for long-term conservation (cryopreservation), without the need to move the animals. Along with cooling systems, dilution media are fundamental to semen processing protocols. To our knowledge, this is the first study to establish protocols for integrating cooling and cryopreservation techniques for peccary semen using shipping containers. Overall, this study revealed promising new alternative protocols for the cryopreservation of peccary semen, opening new perspectives for this technique.

**Abstract:**

We verified the possibility of cooling peccary semen for 4, 24, and 48 h before cryopreservation, using different dilution media (TRIS + egg yolk (20%) and PRIMXcell Ultra). Ten ejaculates were divided equally into six aliquots and then diluted. Two aliquots were stored in a biological incubator (4 h), and the remaining aliquots were stored in a commercial container, the Botutainer^®^ (24 and 48 h), both at 5 °C. The samples were cryopreserved and then evaluated for kinetic parameters, functionality, integrity, mitochondrial activity, morphology, and sperm binding capacity. After thawing, samples diluted in TRIS showed total motility of 43.4 ± 6.8%, 48.4 ± 6.2%, and 38.6 ± 5.0% after cooling for 4, 24, and 48 h before cryopreservation, respectively. Such results are significantly greater than those achieved with the use of PRIMXcell diluent for 4 (8.3 ± 2.8%), 24 (4.7 ± 1.4%), and 48 h (4.8 ± 2.9%) storage (*p* < 0.05). Furthermore, TRIS provided better preservation of sperm membrane integrity when samples were cooled for 24 h (44.5 ± 4.7%) before cryopreservation compared to those samples diluted in PRIMXcell Ultra stored for 24 (25.7 ± 4.0%) and 48 h (25.2 ± 4.0%) before freezing (*p* < 0.05). In summary, we suggest TRIS diluent + egg yolk (20%) as an effective option to allow semen to cool for 24 or 48 h in a transport container before cryopreservation.

## 1. Introduction

Given the persistence of threats to the survival of various free-living species, there is a growing need for efficient strategies to conserve genetic material [1]. The creation and establishment of biological sample repositories (biobanks) are emerging as promising options for the preservation of wild animals, especially for species threatened with extinction [2]. To ensure the successful implementation of biobanks, it is essential to master alternative strategies, with cryopreservation being the key biotechnology [3].

Several studies have focused on the cryopreservation of male gametes from ecologically important individuals such as collared peccaries (*Pecari tajacu* Linnaeus, 1758) [1,4]. These peculiar wild ungulates, originally found in North, Central and South America, are of great ecological importance; however, population fragmentation has occurred in some biomes [5]. Since 2010, protocols for cryopreservation of peccary semen have been developed [6]. Although cryopreservation is the preferred method for preserving semen over an indefinite period, it has several limitations under field conditions. These include the lack of facilities and equipment required for processing and difficulties in obtaining and transporting this valuable biological material [7]. Therefore, alternative procedures or modifications to conventional cryopreservation protocols for peccaries should be developed to ensure sperm preservation, particularly for collection in remote locations [7].

Semen cryopreservation could be improved through the development of specific protocols and equipment for semen cooling [8], allowing transportation and cooling curves different from those used in existing cryopreservation protocols [6]. The cooling storage of semen (between 4 and 5 °C) is an emerging alternative for the preservation of various livestock species; however, it is still largely neglected in free-living specimens [9]. Short-term conditioning (cooling) can be carried out in two different ways: active systems that use automated equipment, which guarantees predetermined and more consistent cooling curves [10], and passive cooling, which is carried out using isothermal boxes (transport devices or Styrofoam boxes) [11]. There are several models of cooling containers on the market for transporting semen including Equitainer^®^ (Hamilton-Thorne Research, Beverly, MA, USA) or Botu-Box^®^ and Botutainer^®^ (Botupharma Biotecnologia Animal, Botucatu, Brazil) [12]. The Botutainer^®^ [12] is a commonly used transportation device in Brazil that has an initial cooling curve of approximately −0.3 °C/min, guaranteeing a final storage temperature of around 5 °C and a maximum storage time of 48 h [12]. The application of transport devices aims to ensure a slow decline in temperature to minimize cold damage to spermatozoa [13,14]. The ability to transport cooling sperm from free-living species allows genetic material to be exchanged between institutions (zoos, reserves, breeding grounds, etc.) and shipped to specialized laboratories for long-term conservation (cryopreservation) without the need to move the animals [8,9].

Along with cooling systems, dilution media are fundamental to semen-processing protocols [15]. To date, in published studies on peccary semen, tris-hydroxymethyl-aminomethane (TRIS)-based diluents supplemented with egg yolk have provided approximately 40% motile sperm for up to 36 h under storage at 5 °C [4,16]. Commercial dilution media are promising for processing cooling semen as they have a chemically defined composition [17]. In domestic swine, a species that is phylogenetically close to the tayassuids [18], some commercial dilution media are routinely used, such as PRIMXcell Ultra^®^ (IMV Technologies, L’Aigle, France), a medium rich in antibiotic complexes, buffering agents, and bioactivating molecules [19]. Preliminary results have demonstrated the efficiency of PRIMXcell Ultra^®^ in maintaining peccary sperm parameters (~67.8% sperm motility and ~74.8% membrane functionality) for up to 48 h under refrigeration at 5 °C [20]. These promising results encouraged us to investigate the effects of this diluent in greater detail.

In this context, this study aimed to verify the possibility of cooling packaging (Botutainer^®^) of collared peccary semen for 4, 24, and 48 h before cryopreservation using different dilution media (TRIS + egg yolk (20%) and PRIMXcell Ultra) to maintain the quality of the sperm after thawing. To the best of our knowledge, this is the first study to integrate cooling and cryopreservation techniques for peccary semen using transport containers.

## 2. Materials and Methods

### 2.1. Animal Ethics and Husbandry

The procedures were approved by the Ethics Committee for the Use of Animals (CEUA) of the Federal University of the Semiarid Region (opinion no. 12/2022) and were authorized by the Chico Mendes Institute for Biodiversity (opinion no. 37329/3). All reagents were purchased from Sigma-Aldrich (St. Louis, MO, USA) and Thermo Fisher Scientific (Carlsbad, CA, USA). The PRIMXcell Ultra^®^ commercial extender was purchased from IMV Technologies (L’Aigle, France).

The animals came from the Wild Animal Multiplication Center (CEMAS—UFERSA, Mossoró, RN, Brazil), located at 5°10′ S and 37°10′ W at an altitude of 16 m, characterized by a semiarid tropical climate. Ten male collared peccaries were selected, all sexually mature and clinically healthy, with an average age of 4.37 ± 1.3 years. The males were divided into three groups and kept in separate paddocks (20 m × 3 m), each with a covered area of 3 m × 3 m, under a natural photoperiod. A balanced diet previously established for this species was provided, consisting of 3300 kcal/kg (isocaloric) and 14% crude protein (isoprotein) and supplemented with tropical fruits. Water was provided ad libitum.

### 2.2. Experimental Design

The experiment was conducted between July and September 2023. The collection, evaluation, and processing of two ejaculates (one per male) per collection day were conducted on a weekly basis. The ejaculates obtained were divided equally into six aliquots, according to the number of storage time intervals used (4, 24, and 48 h) and the number of extenders, with the first aliquots diluted in TRIS (consisting of 3.03 g Tris-hydroxymethyl-aminomethane, 1.78 g monohydrated citric acid, and 1.25 g D-fructose dissolved in 100 mL of ultrapure water and then 20% egg yolk added) + egg yolk (20%) and the others in PRIMXcell Ultra^®^ (prepared according to the manufacturer’s instructions), which were used for cooling and subsequent cryopreservation. For the control group, two aliquots were placed in a biological incubator (Quimis, Diadema, SP, Brazil) at 5 °C and equilibrated for 4 h with a view to the subsequent glycerol addition and cryopreservation stage [6]. The remaining four aliquots were placed in a hermetically sealed transport container system, known as a Botutainer^®^, with a final storage temperature of 5 °C [21]. They remained in this environment for 24 and 48 h in preparation for the subsequent glycerol addition and cryopreservation processes. A final concentration of 100 × 10^6^ sperms/mL was used for both extenders. After cryopreservation, kinetic parameters, membrane functionality and integrity, mitochondrial activity, sperm morphology, and sperm binding capacity were assessed in fresh and frozen/thawed samples.

### 2.3. Semen Collection and Initial Evaluation

After 12 h of food restriction, the males were restrained mechanically using a hand net and intravenously administered propofol (Propovan^®^, Cristalia, Fortaleza, Ceará, Brazil) in bolus (5 mg/kg) [22]. During the procedures, systemic parameters of the heart and respiratory rate were monitored. Semen was obtained using an electroejaculation protocol previously established for the species, using equipment connected to a 12 V supply (Autojac^®^, Neovet, Campinas, SP, Brazil). After asepsis, the animals were placed in the lateral decubitus position and the probe was properly lubricated and inserted into the rectum of the animal with the electrodes positioned ventrally, accompanied by the progressive administration of electrical stimuli. The electrostimulation protocol followed that described by Castelo et al. [6], which consisted of 10 stimuli of 5 V accompanied by an increase in voltage of 1 V until it reached 12 V.

The semen samples obtained were immediately analyzed for volume (micropipetting), pH (pH indicator strips Neutralit^®^, Merck, Bucharest, Romania), and sperm concentration (Neubauer chamber) [22,23]. To assess the sperm morphology, smears were prepared using the wet chamber technique and stained with Bengal Rose stain. The slides were evaluated under a light microscope by counting 200 cells per slide, which were classified as normal or showing morphological defects such as head, midpiece, and tail defects [24].

### 2.4. Procedures for Semen Cooling

Before processing, the semen samples remained in a water bath at 37 °C, the same temperature as the extenders. After diluting the samples in TRIS + egg yolk or PRIMXcell Ultra and immediately placed in a cooling system. The diluted fractions were subjected to two cooling systems: a biological incubator (Quimis, São Paulo, Brazil) with a temperature-controlled system at 5 °C (control group) and a commercial semen transport container (Botutainer^®^, Botupharma, Botucatu, Brazil) at 5 °C, respecting the cooling curves recommended for each device. The temperature of the shipping container was monitored at intervals of 24 and 48 h using a digital temperature gauge with an electronic LCD screen (thermometer with external sensor), which has a temperature range of −50–+110 °C and an accuracy of ± 1 °C. The average temperature values obtained in the evaluated intervals were 6.5 ± 0.7 and 11.6 ± 1.3 °C, respectively.

**Figure 1:**
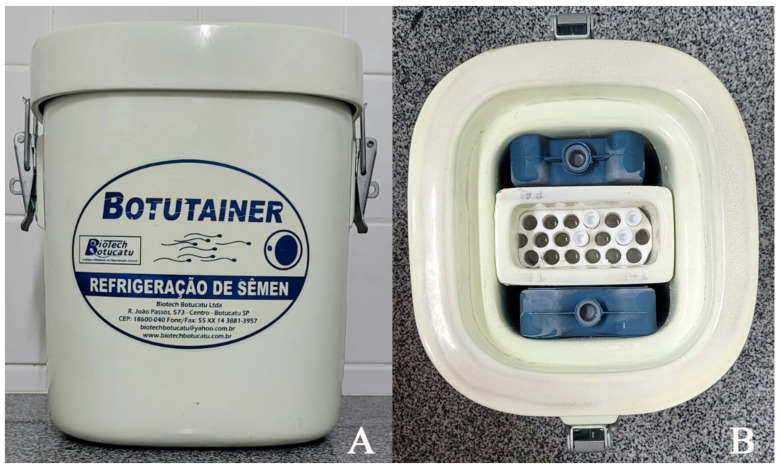
(**A**) Transport container—Botutainer^®^; (**B**) storage of semen samples in the transport container filled with recyclable ice blocks.

The diluted and biologically incubated (control group) conditioned fractions were previously stored in a beaker water jacket (30 mL) at 27 °C and equilibrated for 4 h until they reached 5 °C, as previously described for peccaries by Castelo et al. [6]. The samples destined for the passive semen cooling system (Botutainer^®^) were placed in a specific transport container containing water [25]. Blocks of recyclable ice were then added inside the container, which was hermetically sealed (Figure 1). Scheduled removal was performed at specific intervals of 24 and 48 h, and the removed samples were subjected to cryopreservation.

Immediately after cooling, at the recommended intervals (4, 24, and 48 h), the kinetic parameters of the samples derived from both systems were evaluated to check the post-cooling values and verify whether the samples could be cryopreserved.

### 2.5. Semen Freezing–Thawing Procedures

For the cryopreservation protocol, the cooling samples already diluted in TRIS + yolk (20%) and PRIMXcell Ultra were removed from the respective cooling systems at specific intervals (4, 24, and 48 h), and 6% glycerol was added (also at 5 °C), resulting in a final concentration of 3% glycerol in the extender [4]. The samples were then filled into 0.25 mL straws, which were then placed on a ramp (height 5 cm), exposed to liquid nitrogen for 5 min, and stored in a cryobiological cylinder at −196 °C [1]. After a week, the samples were thawed in a water bath at 37 °C [4], and sperm parameters were assessed.

### 2.6. Computer-Aided Semen Analysis

The kinetic parameters of fresh, cooled, and thawed semen samples were evaluated using a computerized semen analysis system (IVOS 7.4G; Hamilton Thorne Research TM, Beverly, MA, USA) configured according to the definitions previously established for peccaries by Souza et al. [4]. Adjustments were made to the equipment’s setup, modifying the temperature aspects to 37 °C; 60 frames/s; minimum contrast, 45; straightness threshold, 30%; low-speed middle pathway cut-off (VAP), 10 m/s; and average VAP cut-off point, 30 m/s [4]. Samples (~3 µL) were evaluated in 20 µm Leja-4 chambers (IMV Technologies, France). The parameters evaluated were total and progressive motility (%), mean path velocity (VAP; µm/s), straight-line velocity (VSL; µm/s), curvilinear velocity (VCL; µm/s), lateral head amplitude (ALH; µm), beat cross frequency (BCF; Hz), straight linearity (STR, %), and linearity (LIN, %). When there was a low VAP cut-off (LVC) and a medium VAP cut-off (MVC), the overall sperm population was subdivided into four categories: fast, VAP > MVC; medium, LVC < VAP < MVC; slow, VAP < LVC; and static, without cell motility. To ensure the best possible accuracy in measuring the kinematic patterns of semen, the Edit IVOS 7.4 G System Tracks tool was used to manually exclude possible residues derived from the extender. Further dilution in sterile phosphate-buffered solution (PBS) (1:2) was performed when necessary [4].

### 2.7. Sperm Membrane Functionality

A hypoosmotic test was performed to assess the functional integrity of the plasma membrane. Aliquots of fresh and thawed semen were added to a hypo-osmotic solution (distilled water with 0 mOsm/L) and incubated at 37 °C. After the incubation period (45 min), a fraction of the semen was analyzed using phase contrast microscopy (Alttion^®^, Wuzhou, China). A total of 200 cells were counted, and cells with functional membranes were considered to have curled tails, as described by Santos et al. [26]. 

### 2.8. Plasma Membrane Integrity and Mitochondrial Activity

Plasma membrane integrity and mitochondrial activity were assessed simultaneously using a combination of fluorophores. Fresh and/or thawed semen samples (10 μL) were incubated in 5 μL of Hoechst 342 (H-342; Sigma Aldrich, St. Louis, MO, USA) in a dry bath at 37 °C for 8 min. Then, 5 μL of Mito Tracker red^®^ (CMXRos, Molecular Probes, M-7512) and 3 μL of propidium iodide (PI, Sigma Aldrich, Co., St. Louis, MO, USA) were added for a further 10 min. After incubation, the samples were evaluated using an epifluorescence microscope (Episcopic Fluorescent Attachment EFA Halogen Lamp Set, Leica, Kista, Sweden), and 200 cells were counted. Counted sperms were classified as having an intact membrane (head marked in blue with Hoechst 342 (H-342)) or a non-intact membrane (head totally or partially marked in red (PI)). In addition, sperm cells with red markings in the midpiece were considered to have active mitochondrial function, as described by Souza et al. [4].

### 2.9. Sperm Binding Ability Assay

For fresh and frozen–thawed samples, an interaction test with the hen’s egg perivitelline membrane was used to assess the sperm binding capacity, as previously established for peccaries [27]. Briefly, perivitelline membranes obtained from the yolks of fresh unfertilized eggs were used. Initially, the albumen content was drained, followed by wrapping the egg yolk in parafilm to separate the membrane, washing it in saline solution (0.9% NaCl), and standardizing 1 cm^2^ sections, recommending two membranes per treatment. Each perivitelline membrane was then placed individually in well plates filled with the incubation medium. The incubation medium was formulated with the following constituents: phenol red (10 μg / mL), gentamicin sulfate (50 μg / mL), BSA (6 mg / mL), glucose (5.5 mM), sodium pyruvate (0.45 mM), caffeine (1.4 mM), calcium lactate (10 mM), Hepes (10 mM), MgCl_2_ (0.5 mM), CaCl_2_.2H_2_O (2.0 mM), NaCl (114 mM), KCl (3.1 mM), NaH_2_PO_4_ (0.4 mM), and NaHCO_3_ (25 mM) and had a pH of 7.4–7.8. Semen samples (fresh or thawed) were evaluated, incubated in medium (1:1), and centrifuged at 700× *g* for 10 min at room temperature. The floating fraction was discarded, leaving only the sedimented part, which was resuspended in the incubation medium, pre-setting the concentration at 1.0 × 106 motile sperm per mL. The membranes were incubated together with the sperm in a water bath at 38.5 °C for 20 min. Afterward, the sperm were extracted by washing the perivitelline membranes and incubating them in Hoechst 33.250 for 15 min. Finally, the membranes were placed on slides and covered with coverslips for subsequent evaluation and counting of sperm attached to the perivitelline membrane using epifluorescence microscopy (Episcopic Fluorescent Attachment EFA Halogen Lamp Set; Leica, Kista, Sweden) at 400× magnification.

### 2.10. Statistical Analysis

The results are expressed as mean and standard error. The normality of the residuals was verified using the Shapiro–Wilk test, and the homogeneity of variance was verified using the Bartlett test. To meet parametric assumptions, data on progressive motility, mitochondrial activity, STR, LIN, fast, medium, and morphology parameters were subjected to angular transformation [arc-sin (√x/100)] to control possible instabilities.

The semen kinetic parameters obtained after the cooling protocol were analyzed using a linear mixed model (mixed-effects analysis procedure) with repeated measures over time, including the different dilution media, storage intervals, and their interactions as the main effects. Tukey’s and Sidak’s tests were used to compare the means when significant effects were found in the F-test of the analysis of variance.

For the post-thawing results of sperm parameters, kinetics, number of sperms adhered to the membrane, and sperm morphology, a linear mixed model was used (mixed-effects analysis procedure), which considered the measurements as paired data (different aliquots of the same ejaculate), including fixed effects linked to the groups (combined effect of diluents and times) and random effects related to the individualization of each semen sample (each ejaculate). When significant effects were found in the analysis of variance, Tukey’s test was used to compare the means between treatments, and, where appropriate, Dunnett’s test was used to compare the reference group with the other treatments. All analyses were carried out using GraphPad Prism^®^ software version 8 for Windows (GraphPad Software Inc., San Diego, CA, USA), with *p* < 0.05.

## 3. Results

### 3.1. Fresh Semen Parameters

The fresh ejaculates had a milky, whitish appearance and a pH of 6.9 ± 0.2. The average volume obtained was 2.9 ± 0.3 mL, with a sperm concentration of 680.0 ± 68.9 × 106 sperm/mL, of which 94.2 ± 1.6% showed motility and 66.8 ± 4.9% progressive motility. In addition, the fresh samples had 93.7 ± 1.3% functional membranes, 83.0 ± 3.6% viable sperm, 91.8 ± 3.9% mitochondrial activity, and 67.4 ± 3.3% normal sperm. An average of 255.6 ± 24.8 of the sperm were attached to the perivitelline membrane of chicken eggs. Table 1 shows the average, minimum, and maximum values of the sperm and morphological and cytokinetic parameters of the ejaculate of collared peccaries.

### 3.2. Computer-Assisted Semen Analysis in Chilled Samples

The kinetic parameters of the samples were evaluated immediately after cooling (at 4, 24, and 48 h) (Table 2). Under cooling conditions, the total and progressive motility did not differ between the experimental groups (*p* > 0.05). However, progressive motility was reduced over time in the samples diluted in TRIS + egg yolk for the 48 h interval compared to the 4 h interval (*p* < 0.05). Additionally, PRIMXcell Ultra provided better preservation of BCF and STR than the TRIS + egg yolk extender at 24 and 48 h during transport container storage (*p* < 0.05). In addition, significant reductions in BCF and STR parameters were observed over time in both diluents (TRIS + egg yolk (24 and 48 h) and PRIMXcell Ultra (48 h) compared to the initial intervals (*p* < 0.05).

A decrease in medium-velocity sperms was observed in samples diluted in TRIS + egg yolk compared to PRIMXcell Ultra (*p* < 0.05; Table 3). Other sperm subpopulations did not differ between the tested extenders, but slow-velocity sperms increased over time in samples diluted in TRIS + egg yolk (*p* < 0.05).

### 3.3. Post-Thawing Computer-Assisted Semen Analysis

Immediately after thawing, the experimental groups diluted in TRIS + egg yolk and cooled for 4, 24, and 48 h showed the best values for total and progressive motility compared with the experimental groups diluted in PRIMXcell Ultra (*p* < 0.05), as shown in Table 4. In addition, the appearance of ALH in the samples cryopreserved after 48 h of cooling showed that TRIS + egg yolk diluent was the most efficient medium (*p* < 0.05). Finally, samples diluted and cryopreserved in PRIMXcell Ultra after 24 h of cooling showed better preservation of VCL and ALH parameters than those cryopreserved after 4 and 48 h of cooling (*p* < 0.05).

For post-thawing sperm subpopulations, samples diluted in TRIS + egg yolk provided better preservation of fast- and medium-velocity sperm than the groups diluted in PRIMXcell Ultra (*p* < 0.05). In addition, a significant increase in the number of static sperms was observed in all treatments diluted in PRIMXCell Ultra (*p* < 0.05), with a consequent reduction in motile sperms in the fast and medium subpopulations.

### 3.4. Post-Thawing Sperm Membrane and Mitochondrial Activity Analysis

Immediately after thawing (Table 5), both extenders preserved membrane functionality and mitochondrial activity regardless of the duration of previous cooling storage (*p* > 0.05). However, for membrane integrity, samples cooled for 24 h in TRIS + egg yolk extender provided more effective preservation than those cooled for 24 and 48 h in PRIMXcell Ultra diluent (*p* < 0.05).

### 3.5. Post-Thawing Sperm Morphology

After thawing, the TRIS + egg yolk extender provided better preservation (*p* < 0.05) of normal morphology (Table 6) than the PRIMXcell Ultra extender, in which an increase (*p* < 0.05) in sperm defects was observed for samples cooled for 4 and 24 h (*p* < 0.05). Curled and folded tails were the most common morphological defects in all groups tested.

### 3.6. Post-Thawing Sperm Binding Ability

In the sperm binding assay (Table 7), no significant differences were observed between the experimental groups. However, only those samples diluted in TRIS + egg yolk and previously cooled for 24 and 48 h in the Botutainer^®^ presented values for the number of bound sperm similar to those verified for the fresh control group (*p* > 0.05).

## 4. Discussion

Protocols for preserving male gametes in collared peccaries have improved using TRIS-based extenders in recent years [1]; however, the conditions imposed in this experimental trial, using a transport container at 5 °C followed by cryopreservation, represent a new and unprecedented approach to processing peccary semen. For this purpose, we used the cooling protocol previously described for this species, in which semen temperature was equilibrated in a biological incubator at 5 °C for 4 h [6], as a control. This protocol is technically complex to allow semen transportation, but we demonstrated that this obstacle can be easily overcome by using containers like Botutainer^®^, as previously demonstrated for equine [28] and bovine [29].

A slow cooling rate and equilibration time are important stages in the cryopreservation process [30], giving sperm the necessary period to adapt to thermal changes. In this sense, collared peccary semen could be efficiently cooled during transport in a container for up to 48 h using both diluents as both promoted the conservation of sperm motility in values ranging from 65 to 77%. These results are better than those found in the literature, where average values of 40 to 45% total sperm motility were reported for peccary semen stored in TRIS-based extenders for 36 h at 5 °C in a biological incubator [4,16]. A crucial and very important factor in achieving such promising results is the storage temperature. According to Sancho et al. [31], understanding the physiological state reached by porcine spermatozoa at temperatures of 5 °C is essential to explain the survival of sperm cells during the cooling/cryopreservation process, as the cooling storage of semen promotes a reduction in sperm metabolism, significantly saving energy reserves, thus contributing to better preservation and maintenance of the functional aspects of sperm [32]. Additionally, Botutainer^®^ is known for providing a cooling rate (−0.65 °C/min) very similar to those obtained by freezing units (TK 4000^®^, Uberaba, Brazil) for the storage of bovine semen [33].

A finding is that peccary semen can be efficiently packaged (in Botutainer^®^) under cooling conditions for up to 48 h before cryopreservation. The values achieved for post-thawing sperm motility, mainly using TRIS + egg yolk, were in the range commonly reported for the species using conventional protocols of 27.1 ± 5.0 [4] to 41.8 ± 3.5% [1]. Porcine semen cannot be immediately cryopreserved, according to Torres et al. [34]. The authors found that storing porcine semen for 24 h at 17 °C before cryopreservation guaranteed an increase in sperm motility parameters and membrane integrity after thawing compared to the initial intervals. Such statements are also valid for peccary semen, in which storage for 24 or 48 h guaranteed post-thawing sperm quality, even if slight differences were found according to different storage times (4, 24, or 48 h).

In general, the results indicate the better efficiency of TRIS + egg yolk (20%) in preserving peccary kinetic parameters, such as progressive motility, VAP, VCL, ALH, and sperm subpopulations with fast and medium velocities, using different prior cooling times. These kinetic parameters are directly related to the ability of the sperm to fertilize [35]. This efficiency may be related to the basic components of this medium, as TRIS has been used as an efficient pH regulator [36]. Furthermore, the phospholipids present in egg yolk have beneficial binding and protective properties for the plasma membrane [37], acting as non-permeable cryoprotectants [38] and protecting the functional integrity of sperm against thermal shock and cryoinjury [39,40]. However, during its use for the conservation of collared peccary semen, oscillations in the maintenance of progressive sperm motility were observed, probably because of the addition of egg yolk, which contains large particles without complete solubility, which can cause the formation of excessive debris that can interfere with the functional aspects of the semen [41].

There were no differences in sperm membrane functionality and integrity and mitochondrial activity among the extenders overall, except for those samples diluted in TRIS + egg yolk and cooled for 24 h, which presented the most effective preservation of plasma membrane integrity compared to those diluted in PRIMXcell Ultra and stored for 24 and 48 h before cryopreservation. The aforementioned efficiency of the medium in preserving these sperm parameters is mainly due to its components. TRIS has egg yolk as its main functional component, which plays a direct role in defending the plasma membrane against temperature-related damage [42], guaranteeing the maintenance of plasma membrane integrity and protecting against cryolesions [38,43]. For PRIMXcell Ultra, IMV Technologies have developed additives (not detailed) that preserve the main organelles of the sperm, such as mitochondria, acrosome, and plasma membrane, guaranteeing their viability and protecting the integrity of the sperm until oocyte fertilization [19]. The optimized formulation of PRIMXcell Ultra offers remarkable efficiency in energy production and the protection of various functions of spermatozoa [19].

Additionally, both media provided similar values in the sperm binding assay, although the TRIS + egg yolk extender showed values similar to those of the fresh control group. This functional assay is useful for assessing the sperm fertilization potential [41]. Moreover, the use of multiple parameters in the analysis of semen samples, especially those related to motility and cell morphology, favors a better estimation of the fertilizing potential of spermatozoa [44]. In the sperm binding test, the best results were obtained with the TRIS + egg yolk extender, especially with longer refrigeration curves (24 h and 48 h). Similar results were previously observed in the semen of domestic pigs, in which the adoption of cooling curves for up to 24 h, at 10 °C [45] and 15 °C [46], promoted an increase in sperm characteristics after thawing, thus contributing to an increase in the fertilizing potential of the samples. However, the mechanisms by which an increase in the cooling curve contributes to more successful post-thaw results have not yet been fully elucidated. In porcine semen, it has been suggested that a 24 h holding time in diluted semen allows better protection of sperm against heat shock-induced cryolesions by maintaining the architectural arrangement of the lipid molecules in the plasma membrane [47], thus increasing the cryosurvival of the semen. Our results imply similar conclusions for collared peccaries.

In general, PRIMXcell Ultra is not the most viable option for cryopreserving refrigerated samples for 4, 24, and 48 h, especially if samples are intended for artificial insemination programs. Based on the results obtained in the sperm binding assay, it is possible to infer that peccary samples diluted, chilled, and cryopreserved in PRIMXCell^®^ Ultra could be used for in vitro fertilization, but this remains to be confirmed. This statement is supported by the fact that for peccaries, efficient methods of sperm selection using Percoll gradients for in vitro fertilization are already being developed and have been capable of generating heterologous embryos using porcine oocytes [48]. Moreover, this commercial extender contains some non-informed “bioactive” molecules whose main function is to potentiate the biosynthesis of platelet-activating factors (PAFs) [19], which stimulate motility, fertilizing potential, and fertility rates [49]. Additionally, although the post-thawing results were inadequate, we cannot deny the efficiency of the PRIMXCell(R) Ultra in preserving semen from collared peccaries under refrigeration, as previously demonstrated for the same species [20] and domestic swine [19]. Therefore, its use for semen transport cannot be ruled out, although it is necessary to wash the samples later if they are intended for cryopreservation using another extender, such as TRIS + egg yolk, which was the most efficient extender for all stages of the process in the present study.

## 5. Conclusions

Peccary semen was efficiently cooled in a transport container, the Botutainer^®^, for up to 48 h before cryopreservation, without significantly compromising the functional aspects of the semen after thawing. We recommend the use of the TRIS + egg yolk (20%) extender, while the commercial PRIMXcell Ultra medium is not suitable for this purpose.

## Figures and Tables

**Table 1:** Mean (mean ± SEM), minimum, and maximum values of sperm aspects, and the morphological and kinetic parameters of fresh adult collared peccary ejaculates (n = 10) used in the experiment.

Semen Variables	Mean ± SEM	Min	Max
Volume (mL)	2.9 ± 0.3	1.6	4.4
Concentration (×10^6^ sperm/mL)	680.0 ± 68.9	230.0	960.0
pH	6.9 ± 0.2	5.5	7.500
Membrane functionality (%)	93.7 ± 1.3	83.0	98.0
Membrane integrity (%)	83.0 ± 3.6	63.0	94.0
Mitochondrial activity (%)	91.8 ± 3.9	65.0	100.0
**Sperm Morphology**			
Normal morphology (%)	67.4 ± 3.3	53.0	82.0
Acrosome defects (%)	0.6 ± 0.3	0.0	2.0
Normal isolated head (%)	1.4 ± 0.6	0.0	5.0
Abaxial, rectroaxial, and oblique (%)	3.3 ± 0.6	1.0	6.0
Curled or bent tail (%)	16.4 ± 3.0	8.0	41.0
Tightly curled tail (%)	6.0 ± 2.6	0.0	26.0
Proximal cytoplasmic droplets (%)	2.5 ± 0.7	0.0	8.0
Distal cytoplasmic droplets (%)	2.4 ± 0.6	0.0	5.0
**Sperm kinetic parameters**			
Total motility (%)	94.2 ± 1.5	82.0	98.0
Progressive motility (%)	66.8 ± 4.8	41.0	87.0
Velocity average pathway (VAP; µm/s)	67.6 ± 6.7	42.6	97.7
Velocity straight line (VSL; µm/s)	47.9 ± 5.7	29.5	78.4
Curvilinear velocity (VCL; µm/s)	129.8 ± 9.2	84.9	173.3
Amplitude of lateral head (ALH; µm)	6.4 ± 0.3	4.6	7.4
Beat cross frequency (BCF; Hz)	36.2 ± 1.1	31.4	43.6
Straightness (STR; %)	69.3 ± 2.1	60.0	81.0
Linearity (LIN; %)	36.7 ± 2.4	28.0	50.0
**Sperm Subpopulations**			
Rapid (%)	81.4 ± 4.7	56.0	97.0
Medium (%)	12.6 ± 3.4	1.0	31.0
Slow (%)	2.1 ± 0.5	1.0	5.0
Static (%)	3.7 ± 1.1	1.0	13.0

**Table 2:** Kinetic parameters (mean ± SEM) of collared peccary semen samples (n = 10) after cooling storage at 5 °C in a biological incubator (4 h) and in a transport container (Botutainer—24 and 48 h), using the diluents TRIS + egg yolk (TRIS) and PRIMXcell Ultra (PRIM).

Diluent	Time	Motility (%)	Progressive Motility (%)	VAP (μm/s)	VSL (μm/s)	VCL (μm/s)	ALH (μm)	BCF (Hz)	STR (%)	LIN (%)
TRIS	**4 h**	82.5 ± 4.5	54.5 ± 6.2 ^a^	66.4 ± 4.2	43.6 ± 3.6	119.7 ± 11.6	7.4 ± 0.2	31.8 ± 1.1 ^aB^	62.3 ± 2.1 ^a^	30.9 ± 1.3
	**24 h**	79.1 ± 5.4	41.8 ± 5.1 ^ab^	59.6 ± 4.4	34.3 ± 3.3	138.9 ± 9.0	7.1 ± 0.3	28.2 ± 1.0 ^bB^	53.9 ± 1.8 ^bB^	28.6 ± 1.0
	**48 h**	76.7 ± 4.1	37.0 ± 3.0 ^b^	61.6 ± 5.1	33.1 ± 2.3	113.6 ± 17.0	7.1 ± 0.4	28.4 ± 0.9 ^bB^	51.7 ± 1.4 ^bB^	27.8 ± 1.2
PRIM	**4 h**	74.1 ± 7.1	46.7 ± 7.7	57.4 ± 5.7	41.7 ± 5.0	117.2 ± 9.5	6.4 ± 0.4	37.8 ± 0.7 ^aA^	68.7 ± 2.4 ^a^	35.3 ± 2.3
	**24 h**	77.0 ± 6.9	45.2 ± 5.9	55.6 ± 3.1	36.3 ± 2.8	118.8 ± 5.8	6.6 ± 0.3	36.3 ± 1.1 ^abA^	62.1 ± 2.6 ^bA^	31.3 ± 2.0
	**48 h**	65.8 ± 6.3	34.4 ± 6.0	47.8 ± 3.0	31.2 ± 2.8	101.4 ± 5.0	6.3 ± 0.2	34.8 ± 0.9 ^bA^	62.6 ± 2.5 ^bA^	31.2 ± 2.1
Time *p*-value		0.3317	0.0228	0.1384	0.7068	0.0605	0.4696	0.0006	<0.0001	0.0558
Diluent *p*-value		0.2125	0.6876	0.0678	0.8527	0.3105	0.0607	<0.0001	0.0022	0.0826
Diluent × Time *p*-value		0.6963	0.5567	0.3987	0.7558	0.4710	0.6137	0.4225	0.4287	0.6974

Lowercase letters (^a, b^) indicate a significant difference in measurements over time (4, 24, and 48 h) (Tukey’s test; *p* < 0.05). Capital letters (^A, B^) indicate a significant difference in diluent groups within the same time interval (for example, TRIS vs. PRIM at the 4 h interval) (Sidak test; *p* < 0.05).

**Table 3:** Sperm subpopulations (mean ± SEM) of collared peccary semen samples (n = 10) after cooling storage at 5 °C in a biological incubator (4 h) and in a transport container (Botutainer—24 and 48 h), using the diluents TRIS + egg yolk (TRIS) and PRIMXcell Ultra (PRIM).

Diluent	Time	Rapid (%)	Medium (%)	Slow (%)	Static (%)
TRIS	**4 h**	72.4 ± 5.9	10.1 ± 1.6	3.2 ± 0.5 ^b^	14.3 ± 4.1
	**24 h**	69.4 ± 6.2	9.7 ± 1.2	4.3 ± 0.7 ^ab^	16.8 ± 5.0
	**48 h**	67.5 ± 4.8	9.2 ± 1.3 ^B^	5.2 ± 0.9 ^a^	18.2 ± 3.6
PRIM	**4 h**	58.7 ± 8.6	15.6 ± 3.2	4.0 ± 0.8	21.5 ± 6.8
	**24 h**	62.7 ± 7.1	14.5 ± 1.9	5.3 ± 0.5	19.2 ± 6.4
	**48 h**	48.1 ± 6.7	17.8 ± 2.5 ^A^	4.0 ± 0.6	28.8 ± 6.2
Time *p*-value		0.2741	0.7226	0.0139	0.4648
Diluent *p*-value		0.0653	0.0142	0.7929	0.1970
Diluent × Time *p*-value		0.5360	0.5147	0.5933	0.7257

Lowercase letters (^a, b^) indicate a significant difference in measurements over time (4, 24, and 48 h) (Tukey’s test; *p* < 0.05). Capital letters (^A, B^) indicate a significant difference in diluent groups within the same time interval (for example, TRIS vs. PRIM at the 4 h interval) (Sidak test; *p* < 0.05).

**Table 4:** Post-thawing kinetic parameters (mean ± SEM) of collared peccary semen samples (n = 10) cryopreserved after cooling at 5 °C for 4, 24, and 48 h using TRIS + egg yolk (TRIS) and PRIMXcell Ultra (PRIM) extenders.

Parameters	Frozen–Thawed Semen					
	4 h		24 h		48 h	
	TRIS	PRIM	TRIS	PRIM	TRIS	PRIM
Total motility (%)	43.4 ± 6.8 ^a^	8.3 ± 2.8 ^b^	48.4 ± 6.2 ^a^	4.7 ± 1.4 ^b^	38.6 ± 5.0 ^a^	4.8 ± 2.9 ^b^
Progressive motility (%)	22.6 ± 4.4 ^a^	3.9 ± 1.8 ^b^	21.9 ± 3.6 ^a^	2.5 ± 0.9 ^b^	16.7 ± 2.9 ^a^	2.0 ± 1.4 ^b^
Velocity average pathway (µm /s)	44.3 ± 3.3 ^ab^	26.8 ± 5.2 ^b^	46.6 ± 3.0 ^a^	31.9 ± 5.1 ^ab^	40.6 ± 3.2 ^ab^	26.4 ± 6.9 ^b^
Velocity straight line (µm /s)	28.8 ± 2.5	20.4 ± 4.3	27.1 ± 2.0	22.9 ± 3.3	23.7 ± 2.1	20.3 ± 5.2
Velocity curvilinear (µm /s)	93.9 ± 5.9 ^ab^	56.4 ± 10.6 ^b^	102.5 ± 6.1 ^a^	67.8 ± 13.2 ^ab^	91.9 ± 6.2 ^ab^	52.4 ± 14.8 ^b^
Amplitude lateral head (µm/s)	5.9 ± 0.3 ^ab^	3.5 ± 0.9 ^bc^	6.5 ± 0.3 ^a^	4.1 ± 0.9 ^abc^	6.0 ± 0.3 ^ab^	2.5 ± 0.9 ^c^
Beat cross frequency (Hz)	32.2 ± 0.4	24.1 ± 4.8	31.6 ± 0.8	27.8 ± 6.8	32.1 ± 0.7	21.6 ± 5.8
Straightness (%)	62.6 ± 1.9	56.8 ± 9.9	56.1 ± 1.3	67.9 ± 8.9	57.0 ± 2.3	60.7 ± 10.4
Linearity (%)	30.6 ± 1.3	29.5 ± 5.6	26.2 ± 0.7	40.9 ± 9.6	26.2 ± 1.5	37.4 ± 6.9
Sperm subpopulations						
Rapid (%)	30.5 ± 5.8 ^a^	4.7 ± 1.9 ^b^	35.0 ± 5.1 ^a^	2.9 ± 1.0 ^b^	26.2 ± 4.1 ^a^	2.5 ± 1.6 ^b^
Medium (%)	12.8 ± 1.6 ^a^	3.4 ± 1.1 ^b^	13.3 ± 1.9 ^a^	1.5 ± 0.5 ^b^	12.9 ± 2.1 ^a^	2.2 ± 1.3 ^b^
Slow (%)	3.2 ± 0.4	2.2 ± 0.9	3.6 ± 0.6	1.4 ± 0.8	3.3 ± 0.4	2.4 ± 1.3
Static (%)	53.4 ± 7.0 ^b^	89.5 ± 3.5 ^a^	47.9 ± 6.5 ^b^	94.0 ± 1.8 ^a^	58.0 ± 5.3 ^b^	92.8 ± 3.9 ^a^

^a,b,c^ Lowercase letters superscripted in the lines indicate significant differences between the different experimental groups (Tukey test; *p* < 0.05).

**Table 5:** Values (mean ± SEM) for sperm membrane functionality, membrane integrity, and mitochondrial activity of collared peccary semen (n = 10) diluted in TRIS + egg yolk and in PRIMXcell Ultra, cooled for 4, 24, and 48 h at 5 °C, and subsequently cryopreserved.

Sperm Parameters	Frozen–Thawed Semen					
	4 h		24 h		48 h	
	TRIS	PRIM	TRIS	PRIM	TRIS	PRIM
Membrane functionality (%)	66.6 ± 4.6	60.1 ± 5.9	64.6 ± 3.4	60.9 ± 6.0	55.3 ± 3.8	55.3 ± 6.7
Membrane integrity (%)	39.60 ± 5.6 ^ab^	32.10 ± 4.9 ^ab^	44.5 ± 4.7 ^a^	25.7 ± 4.0 ^b^	35.5 ± 5.2 ^ab^	25.2 ± 4.0 ^b^
Mitochondrial activity (%)	57.0 ± 10.0	49.5 ± 10.6	51.3 ± 8.7	38.4 ± 9.2	45.9 ± 9.5	46.8 ± 9.4

^a,b^ Lowercase letters superscripted in the lines indicate significant differences between the different experimental groups (Tukey test; *p* < 0.05).

**Table 6:** Mean values (mean ± SEM) for morphological analysis of collared peccary (n = 10) semen after freezing–thawing of samples diluted in TRIS + egg yolk and PRIMXcell Ultra, previously subjected to cooling for 4, 24, and 48 h at 5 °C.

Sperm Morphology	Frozen–Thawed Semen					
	4 h		24 h		48 h	
	TRIS	PRIM	TRIS	PRIM	TRIS	PRIM
Normal morphology (%)	52.2 ± 4.9 ^a^	29.0 ± 6.5 ^b^	47.7 ± 5.4 ^a^	33.3 ± 7.6 ^b^	47.2 ± 5.8 ^a^	27.2 ± 7.5 ^b^
Acrosome defects (%)	2.8 ± 0.4	2.6 ± 0.5	2.3 ± 0.5	2.9 ± 0.9	1.8 ± 0.5	2.4 ± 0.7
Normal isolated head (%)	1.3 ± 0.5	2.5 ± 0.8	2.0 ± 0.5	1.4 ± 0.4	1.6 ± 0.5	2.1 ± 0.5
Abaxial, rectroaxial, and oblique (%)	4.0 ± 0.8	5.2 ± 1.6	3.2 ± 0.5	3.0 ± 0.8	3.2 ± 0.8	3.3 ± 0.9
Curled or bent tail (%)	29.2 ± 5.7 ^c^	45.2 ± 8.5 ^ab^	35.4 ± 6.9 ^bc^	49.1 ± 9.6 ^ab^	38.4 ± 6.9 ^ac^	51.7 ± 8.6 ^a^
Tightly curled tail (%)	4.3 ± 2.3	5.5 ± 3.4	3.6 ± 1.9	3.5 ± 1.5	1.6 ± 0.9	1.6 ± 0.6
Proximal cytoplasmic droplets (%)	4.3 ± 1.1	6.9 ± 1.4	3.8 ± 0.9	5.3 ± 1.1	4.3 ± 1.2	7.9 ± 1.4
Distal cytoplasmic droplets (%)	1.9 ± 0.3	3.1 ± 0.9	2.0 ± 1.1	1.5 ± 0.6	1.9 ± 0.8	3.8 ± 1.3

^a,b,c^ Lowercase letters superscripted in the lines indicate significant differences between the different experimental groups (Tukey test; *p* < 0.05).

**Table 7:** Mean values (±SEM) and their range (min–max) for the number of sperm bound to the perivitelline membrane of chicken egg yolk in samples of collared peccary frozen–thawed semen (n = 10) previously diluted in TRIS + egg yolk and PRIMXcell Ultra and cooled for 4, 24, and 48 h at 5 °C before cryopreservation.

Number of Bound Sperm	Fresh	Frozen–Thawed Semen					
		4 h		24 h		48 h	
		TRIS	PRIM	TRIS	PRIM	TRIS	PRIM
Mean ± SEM	255.6 ± 24.8 ^A^	160.2 ± 26.5 ^B^	139.0 ± 23.4 ^B^	209.4 ± 26.5 ^A^	156.6 ± 17.8 ^B^	204.5 ± 24.9 ^A^	176.9 ± 17.9 ^B^
Min	74.8	58.5	37.2	98.3	76.8	77.2	93.0
Max	389.5	367.0	261.7	341.5	257.8	314.3	280.3

^A,B^ Superscript letters indicate a significant difference between the fresh group and the thawed groups (Dunnett test; *p* < 0.05). There were no significant differences between the experimental groups (Tukey test; *p* > 0.05).

## Data Availability

The data presented in this study are available on request from the corresponding author.
